# Benchmarking B-Cell Epitope Prediction with Quantitative Dose-Response Data on Antipeptide Antibodies: Towards Novel Pharmaceutical Product Development

**DOI:** 10.1155/2014/867905

**Published:** 2014-05-11

**Authors:** Salvador Eugenio C. Caoili

**Affiliations:** Department of Biochemistry and Molecular Biology, College of Medicine, University of the Philippines Manila, Room 101, Medical Annex Building, 547 Pedro Gil Street, Ermita, 1000 Manila, Philippines

## Abstract

B-cell epitope prediction can enable novel pharmaceutical product development. However, a mechanistically framed consensus has yet to emerge on benchmarking such prediction, thus presenting an opportunity to establish standards of practice that circumvent epistemic inconsistencies of casting the epitope prediction task as a binary-classification problem. As an alternative to conventional dichotomous qualitative benchmark data, quantitative dose-response data on antibody-mediated biological effects are more meaningful from an information-theoretic perspective in the sense that such effects may be expressed as probabilities (e.g., of functional inhibition by antibody) for which the Shannon information entropy (SIE) can be evaluated as a measure of informativeness. Accordingly, half-maximal biological effects (e.g., at median inhibitory concentrations of antibody) correspond to maximally informative data while undetectable and maximal biological effects correspond to minimally informative data. This applies to benchmarking B-cell epitope prediction for the design of peptide-based immunogens that elicit antipeptide antibodies with functionally relevant cross-reactivity. Presently, the Immune Epitope Database (IEDB) contains relatively few quantitative dose-response data on such cross-reactivity. Only a small fraction of these IEDB data is maximally informative, and many more of them are minimally informative (i.e., with zero SIE). Nevertheless, the numerous qualitative data in IEDB suggest how to overcome the paucity of informative benchmark data.

## 1. Introduction

Antibody-mediated immunity provides the basis for developing novel pharmaceutical agents according to a paradigm whereby such agents are developed in tandem with their prospective antidotes, thus addressing concerns over human safety in a proactive manner that is more acceptable from a regulatory standpoint [1]. In particular, antibodies may be produced against virtually any pharmaceutical agent (e.g., a small-molecule drug or a biological such as a cytokine or even another antibody), such that the antibodies may be useful as antidotes to the agent by virtue of their capacity to neutralize its pharmacologic activity. Furthermore, antidotes may also be developed as catalytic antibodies (i.e., abzymes) produced against transition-state analogs for degradative (e.g., hydrolytic) reactions of specific molecular targets, such that the relevant transition states are stabilized upon binding by the catalytic antibodies, thereby thermodynamically favoring accelerated target degradation [2].

More generally, antibody-mediated immunity comprises an exquisitely rich variety of immune effector mechanisms [3] that can potentially contribute to the control and prevention of infectious and noninfectious clinical conditions (e.g., with exogenously supplied antibodies for passive immunization or endogenous antibodies whose production is induced via active immunization using vaccines). Yet, at least some of the said effector mechanisms can function maladaptively to produce deleterious effects (e.g., antibody-dependent enhancement of infection [4] and hypersensitivity reactions of allergic or autoimmune nature). Such deleterious effects are extremely challenging to predict due to the inherent complexity of immune function in vivo. Hence, any antibody produced for prophylactic or therapeutic purposes (e.g., even only as an antidote to another pharmaceutical agent) must itself be regarded as a potentially hazardous agent to which corresponding antidotes may be developed (e.g., in the form of either anti-idiotypic antibodies or paratope-blocking haptens such as peptide fragments of protein targets).

Among the various pharmaceutical agents, peptidic (i.e., peptide and protein) species are especially advantageous. As regards their manufacture, they are amenable to production via biotechnological as well as synthetic chemical means, with the latter becoming more practically feasible for increasingly longer polypeptide chains [5]. In terms of biotransformation, they are typically metabolized in vivo via main-chain peptide-bond hydrolysis [6], which is less problematic than the metabolism of more exotic xenobiotics that yields toxic metabolites [7]. Moreover, they are potential targets for binding by antipeptide antibodies obtained via immunization with peptide-based immunogens (e.g., vaccines), in which case the antibodies may serve as antidotes to their targets if they neutralize the pharmacologic activity of the said targets upon binding or consequent to downstream immune effector mechanisms.

Where proteins are intended targets of cross-reactive binding by antipeptide antibodies (e.g., to produce antidotes to protein pharmaceutical agents or to elicit protective immune responses against protein virulence factors of pathogens), the immunizing peptides may be designed to contain sequences mimicking B-cell epitopes (i.e., structural features that potentially can be bound by immunoglobulin) on the proteins. Typically, the said sequences are subsequences of the proteins, and they must be both physically accessible for binding by antibodies (e.g., on surface-exposed loops rather than buried within protein interiors) and in conformational states amenable to recognition by corresponding antipeptide paratopes. In principle, such sequences may be identified via B-cell epitope prediction, which entails computational analysis of protein sequences or structures. However, currently available tools for B-cell epitope prediction are of limited utility, especially where the goal is to produce antipeptide antibodies that cross-react with proteins and thereby impact biological function (e.g., by neutralizing protein biological activity). This problem is largely due to key unresolved issues relating to the benchmarking of methods for B-cell epitope prediction [8–10].

To advance B-cell epitope prediction, consensus on benchmark datasets and benchmarking procedures is fundamental [11]. Reaching such consensus remains an open problem, although epistemic inconsistencies clearly can arise from casting epitope prediction as binary classification of submolecular structures (e.g., peptide sequences) into dichotomous (i.e., epitope and nonepitope) categories, notably in the context of peptide-based vaccine design [9, 10]. The potential for these inconsistencies exists where experimental epitope data are curated as either positive or negative, as is the case for all such data in the Immune Epitope Database (IEDB) [12]. On the basis of data thus curated, benchmark datasets are typically created by dichotomously classifying peptide sequences as either epitopes or nonepitopes, such that each identified epitope is associated with at least some experimental data curated as positive (i.e., deemed consistent with binding by antibody). Inconsistency can therefore arise when new experimental data associated with a peptide sequence previously classified as a nonepitope are curated as positive, prompting its reclassification as an epitope; consequently, a benchmark dataset comprising the peptide sequence would be altered by the reclassification, such that benchmarking of epitope-prediction tools before and after the change could yield divergent results.

Still, binary classification continues to dominate as the conceptual basis for B-cell epitope prediction in literature [13, 14]. Apart from the historical dominance of binary classification since the initial published attempts at B-cell epitope prediction [15], at least two other possible explanations may account for the status quo. First, the preponderance of qualitative rather than quantitative benchmark data justifies binary classification on practical grounds, especially considering that quantitative data can be reduced to qualitative data by dichotomization with cutoff values (although this entails loss of information and related problems [16]). Second, an appropriate theoretical framework has yet to be articulated for benchmarking B-cell epitope prediction against quantitative data. The present work thus aims to outline such a theoretical framework and, in light thereof, subsequently examine currently available experimental data from which benchmark datasets might be assembled.

## 2. Theory and Methods

### 2.1. Interpretation of Quantitative Data

In the context of B-cell epitope prediction, the interpretation of empirical data has been complicated by disagreement among investigators as to what constitutes meaningful antibody-antigen binding interaction [18–20]. Immunogenicity of an antigen in the sense of capacity to elicit production of specific antibodies (i.e., which preferentially bind to the antigen and possibly other structurally related targets) implies the existence of at least one B-cell epitope on the antigen. Yet, published work on B-cell epitope prediction has focused mainly on immunogenicity that leads to production of antibodies cross-reactive with (i.e., also capable of preferentially binding) a target other than the immunogen (i.e., antigen used for immunization). Moreover, the bulk of the work has been confined to cross-reactions either of antipeptide antibodies with proteins or of antiprotein antibodies with peptides [9, 21, 22]. The work on antipeptide antibodies is relevant to the development of peptide-based immunogens both for active antibody-mediated immunization (e.g., as peptide-based vaccines) and to produce antibodies either for passive immunization (e.g., for antibody-mediated immunoprophylaxis or immunotherapy) or for immunodiagnostics based on antigen detection. The work on antiprotein antibodies is relevant to the development of peptide-based antigens for immunodiagnostics based on antibody detection. As cross-reactions involving either antipeptide or antiprotein antibodies potentially entail binding of denatured protein [18–20], empirical data on such cross-reactions are difficult to interpret with regard to biological meaning unless these data reflect functional correlates of antibody-antigen binding. However, this problem is overcome for antipeptide antibodies shown to cross-react with a biologically active target in a functional assay (e.g., for enzyme inhibition or pathogen neutralization) [9, 10]. Hence, the present work has been developed primarily with reference to such functionally relevant cross-reactivity, which is nonetheless of great interest from the standpoint of antibody-mediated immunity. Emphasis herein is placed on the aspect of cross-reactivity rather than immunogenicity per se, considering that the latter often can be realized in practice (e.g., through conjugation of peptides with immunogenic carriers and coadministration of the resulting peptide-carrier conjugates with suitable adjuvants).

Benchmarking of published B-cell epitope prediction methods [10, 44] typically employs performance measures that are only indirectly applicable to quantitative benchmark data by way of applying cutoff values (either explicitly set by investigators or implicitly determined by detection limits of laboratory assays) to dichotomize such data and thereby yield corresponding qualitative benchmark data. However, such use of cutoff values incurs the cost of potentially severe information loss and its consequences (e.g., loss of statistical power) [16]. The prediction methods themselves typically generate continuous numerical output that is dichotomized for direct comparison with the qualitative benchmark data, which is commonly accomplished by evaluating the area under the receiver operator characteristic curve (AUROCC or *A*_ROC_) [11]. As an alternative to dichotomizing benchmark data and predictions, these can be used directly as continuous variables to evaluate a performance measure such as the Pearson correlation coefficient (PCC) [9, 10].

For two continuous variables *X* and *Y* of which paired values (*X*_*i*_, *Y*_*i*_) define *n* data points, the PCC (denoted by *r*) can be generalized as a weighted PCC (wPCC), such that

(1) $$\begin{array}{c} r=\frac{\sum_{i=1}^{n}w_{i}\left(X_{i}-\bar{X}\right)\left(Y_{i}-\bar{Y}\right)}{\sqrt{\sum_{i=1}^{n}w_{i}{\left(X_{i}-\bar{X}\right)}^{2}\sum_{i=1}^{n}w_{i}{\left(Y_{i}-\bar{Y}\right)}^{2}}}, \end{array}$$

where *w* is a nonnegative weight while $\bar{X}$ and $\bar{Y}$ are weighted arithmetic means both of the form

(2) $$\begin{array}{c} \bar{Z}=\frac{\sum_{i=1}^{n}w_{i}Z_{i}}{\sum_{i=1}^{n}w_{i}}, \end{array}$$

where *Z* is a generic continuous variable. If the values of *X* are empirically obtained while those of *Y* are corresponding computational predictions, each data point (*X*_*i*_, *Y*_*i*_) may be assigned a weight *w*_*i*_ representing the appraised worth of *X*_*i*_ relative to other values of *X*, such that zero weight is assigned to data points deemed completely worthless while progressively more positive weights are assigned to other data points of increasing appraised worth. The weight thus could be defined as a function of both measurement quality (e.g., as regards accuracy and precision) and informativeness (i.e., the potential usefulness of a particular empirically obtained numeric value in the benchmarking of predictions). For simplicity, the present work focuses mainly on informativeness to define an upper limit on the weight assuming maximum measurement quality (e.g., perfect accuracy and precision).

To facilitate the utilization of benchmark datasets comprising continuous dose-response data on antibody-mediated modulation of biological activity, such data typically can be normalized to yield quotients in the range of zero to unity that represent the magnitude of an observed antibody-mediated biological effect relative to its theoretical or empirically determined maximum magnitude [9, 10]. Each quotient may thus be obtained as

(3) $$\begin{array}{c} q=\frac{B}{B_{0}}, \end{array}$$

where *B* and *B*_0_ are the observed and maximum magnitudes of the antibody-mediated biological effect, respectively. For antibody-mediated inhibition of biological activity (e.g., enzyme catalytic activity or pathogen infectivity), *B* may be equated with the observed fractional activity loss due to binding by antibody, such that *B*_0_ is unity (corresponding to complete loss of activity). Likewise, for antibody-mediated host protection against lethal challenge (e.g., with a toxin or pathogen), *B* may be equated with the observed fractional host survival (i.e., proportion of surviving hosts) due to binding by antibody (e.g., antitoxin or pathogen-neutralizing antibody), such that *B*_0_ is again unity (corresponding to complete protection against lethality).

More generally, *B* and *B*_0_ are readily defined where *q* can be interpreted as the probability of a particular functional state (e.g., catalytically active versus inactive, or viable versus nonviable). In the mechanistically simplest cases, this functional state directly corresponds to the binding state (i.e., either free or antibody-bound) of the antigen of interest (e.g., an enzyme with a single catalytic site that is active in the free state but completely inactivated in the antibody-bound state). In such cases, the probability of the functional state is equivalent to the fraction of antigen that is either free or antibody-bound, with the equilibrium value of the antibody-bound fraction approximated under conditions of antibody excess relative to the antigen by

(4) $$\begin{array}{c} f=\frac{1}{1+\left(K_{D}/\left[\text{Ab}\right]\right)}, \end{array}$$

where *K*_*D*_ is the dissociation constant and [Ab] is the antibody concentration, such that *K*_*D*_ is the value of [Ab] at which half of the binding sites for antibody are occupied. For extension of applicability to more complex cases where cooperative binding interactions occur, (4) may be generalized to a form of the Hill equation [45, 46]:

(5) $$\begin{array}{c} f=\frac{1}{1+{\left(K_{D}/[\text{Ab}]\right)}^{n}}, \end{array}$$

where *n* is an interaction coefficient whose value is a lower bound on the number of binding sites for antibody on the antigen. Equation (5) is similar in form to a phenomenological one [47] that relates the probability *p* of a particular biological outcome (e.g., lethality or infection) to the corresponding dose of causative agent (e.g., toxin or pathogen), as

(6) $$\begin{array}{c} p=\frac{1}{1+{\left(C_{m}/C\right)}^{b}}, \end{array}$$

where *b* is an empirical coefficient, *C* is the dose of causative agent, and *C*_*m*_ is the value of *C* at which *p* is half-maximal, such that *C*_*m*_ is a median effective dose. *C*_*m*_ thus tends to produce the biological effect in half the members of a given test population (e.g., of whole organisms or of cells in vitro) and may, for example, represent the median lethal dose (LD_50_) or the median infectious dose (ID_50_) where the biological effect is lethality or infection, respectively. In cases where *C* represents the concentration of causative agent (e.g., in the medium of a cell culture), *C*_*m*_ is the median effective concentration which, for instance, would be the median lethal concentration (LC_50_) if lethality were the biological effect of interest.

Taken together, (4) through (6) provide means to predictively estimate the empirical quotient *q* in (3) as applied to various types of antibody-mediated biological effects on the basis of antibody concentrations in conjunction with antigen-antibody dissociation constants (which can be estimated from free energy changes of binding that are approximated from structural-energetic analysis of antigens [8, 9, 48]). For instance, where binding equilibrium between causative agent and antibody is attained by preincubation and any subsequent shifts in antigen-antibody binding equilibrium (e.g., resulting from dilution) occur at negligibly slow rates during an assay for biological activity of the causative agent, the concentration *C* of free (i.e., unbound) causative agent might be estimated from the total concentration *C*_0_ of free and antibody-bound causative agent combined. More specifically, *C* thus might be estimated as

(7) $$\begin{array}{c} C=\frac{C_{0}}{1+\left[\text{Ab}\right]/\left(K_{D}\right)}, \end{array}$$

according to (4), for use with (6) (correcting for any preassay dilution) to evaluate *p* as a predictive estimate of the empirical quotient *q* in (3).

Granted that (4) through (7) may be applicable only to relatively simple cases, they nonetheless illustrate the importance of antibody concentration [Ab] in the rendering of predictions that are to be benchmarked against continuous dose-response data normalized as the empirical quotient *q* according to (3). In particular, values of *q* approaching either zero or unity correspond to extremes of [Ab] (i.e., low or high values of [Ab] with negligible or near-maximal biological effects, respectively) and are thus relatively uninformative, insofar as estimation of *q* (e.g., using (4) through (7)) becomes insensitive to variation in [Ab] in the limit of low or high [Ab]. Conversely, the most informative value of *q* is half unity, which corresponds to the point of maximal sensitivity to variation in [Ab] (e.g., at which the second derivative of *f* in (4) and (5) is zero) in the estimation of *q*.

Returning to the problem of assigning the weight *w* per data point for (1) in light of the immediately preceding considerations, if *X* is equated with the empirical quotient *q* in (3) while *Y* is obtained as a predictive estimate of *q* (e.g., by means of (4) through (7)), *w* should be maximal where *q* is half unity and zero where *q* is either zero or unity. These constraints are satisfied by the Shannon information entropy [49] calculated in bits as

(8) $$\begin{array}{c} H=-\left(q\log_{2}q+\left(1-q\right)\log_{2}\left(1-q\right)\right), \end{array}$$

assuming two possible alternative states of the mathematically modeled system (e.g., an enzyme that is either active when free or inactivated when antibody-bound, or a cell that has either survived or died following challenge with a toxin). If the values of *q* (i.e., benchmark data) under consideration are all of maximum measurement quality, *w* may be equated with *H*; otherwise, *w* may be assigned a value less than *H* according to limitations of measurement quality (e.g., of accuracy and precision). In other words, *H* may be regarded as an upper bound on *w* in the limit of perfect measurement quality.

Further clarification is warranted regarding the choice of *H* as a measure of informativeness in the present work considering that *H* has long been recognized instead as a measure of uncertainty, particularly in line with the view of statistical mechanics as an application of information theory [50, 51]. This view holds that uncertainty may be quantitatively expressed as *H* in terms of a probability distribution for the occupancy of microscopic states available to a thermodynamic system, following the form of (8) for a two-state system. Accordingly, the uncertainty is least if occupancy of exactly one state is completely certain (i.e., with probability equal to unity, corresponding to zero entropy), whereas the uncertainty is greatest for a uniform probability distribution over all the available states (i.e., with all states being equiprobable, e.g., having a probability of half unity for each state in a two-state system). The notion of entropy as uncertainty may be extended to systems for which the states under consideration are mutually exclusive outcomes (e.g., death or survival), such that completely certain outcomes are associated with zero entropy while maximally uncertain (i.e., equiprobable) outcomes are associated with maximum entropy (e.g., one bit for two equiprobable outcomes). From the standpoint of predictively estimating the empirical quotient *q* in (3), zero and maximum entropy, respectively, correspond to the most and least trivial predictive tasks in that tolerance for error (e.g., in the estimation of the dissociation constant *K*_*D*_ for use in (4) through (7)) increases without bound as *q* approaches either zero or unity. At these extreme values, *q* thus becomes completely uninformative for benchmarking (consistent with the use of *H* as the weight *w* in (1) and (2)).

### 2.2. Retrieval and Processing of Epitope Data

To explore the availability of quantitative dose-response data for benchmarking B-cell epitope prediction in accordance with the preceding Section 2.1, published data were sought on biological effects mediated by antipeptide antibodies. Accessing IEDB via its web interface (with main URL http://www.immuneepitope.org/) [52, 53], database records containing relevant curated data were retrieved from IEDB through a search conducted using its B Cell Search facility (URL http://www.iedb.org/advancedQueryBcell.php/). Each record thus retrieved pertains to an individual B-cell assay and contains multiple data fields, of which several are defined in relation to the key concepts of “Type” (i.e., epitope type with respect to chemical nature) and “1st Immunogen” (i.e., immunogen initially administered to elicit the production of antibodies).

**Figure 1:**
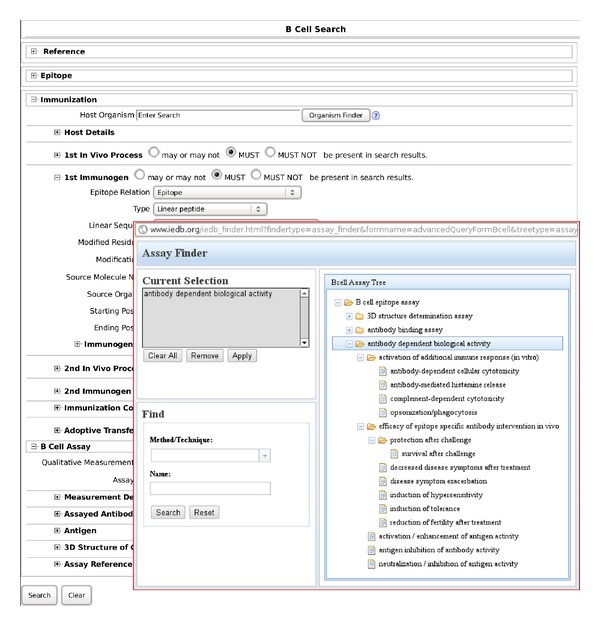
IEDB B-cell search interface (URL http://www.immuneepitope.org/advancedQueryBcell.php/) for retrieval of B-cell assay records. Unless otherwise specified, default options are set. Under “1st Immunogen” (with radio/check button checked for “MUST be present in search results”), “Epitope relation” and “Type” are set to “Epitope” and “Linear peptide,” respectively. Inset with red border contains screenshot of Assay Finder pop-up window (activated by clicking the Assay Finder button located behind inset on “Assay” line, just below “Quantitative measurement” line under “B Cell Assay” heading visible to the left of inset), with “antibody dependent biological activity” selected using the B cell Assay Tree (shown in right panel of inset).

The search was restricted by specifying values for the data fields named “Type” and “1st Immunogen Epitope Relation” (hereafter referred to as the epitope-type and immunogen fields, resp.). The search was performed with the epitope-type and immunogen fields having values set to “Linear peptide” and “Epitope,” respectively. Additionally, the search was further restricted to only those records containing data on antibody-mediated biological effects, which was accomplished by filtering with respect to B-cell assay type (on the data field named “Method/Technique”). Such filtering was performed using the Assay Finder feature of the B Cell Search facility. Within the Assay Finder pop-up window, the B cell Assay Tree was navigated to view the available assay-type categories under the heading of “antibody dependent biological activity,” clicking this heading to select all of the pertinent B-cell assay types (Figure 1).

The search was thus conducted on 18 February 2014, and the search results were downloaded as an IEDB full-format comma-separated value (CSV) file comprising B-cell assay records, which was accessed using the Gnumeric version 1.10.8 GNOME spreadsheet application. Subsequent processing of records focused primarily on the data field named “Quantitative measurement” whose numeric value (where actually specified) corresponds to a measurement of some antibody-mediated biological effect. Records wherein this data field was empty were excluded from further consideration for quantitative analysis, as were others wherein the data field named “Measurement Inequality” contained an inequality symbol (either “<” or “>”, indicating that the numeric value was a lower or upper bound rather than a point estimate). For each record that was ultimately retained for quantitative analysis, the numeric value was compared with that originally reported in the underlying literature reference to check for consistency and was subsequently used to compute the corresponding Shannon information entropy values by means of (3) and (8).

In addition, the entire set of retrieved records was analyzed from a broader perspective to obtain positive- and negative-data record counts for the various B-cell assay types, with special attention to the data field named “Assay Type Units” (which indicates the units of measurement for the result of the B-cell assay, in cases where such units actually have been specified). This was performed in order to assess the potential for capturing quantitative data within the existing IEDB framework, considering that the qualitative B-cell assay data are based on quantitative or potentially quantifiable outcomes.

## 3. Results and Discussion

### 3.1. Curated Quantitative Data

A total of 3996 records on biological effects mediated by antipeptide antibodies was retrieved from IEDB via the performed search, but, of the said records, a subset of only 43 (Table 1) was found to contain explicitly specified numeric values of curated quantitative data representing point estimates of the measured biological effects.

**Figure 2:**
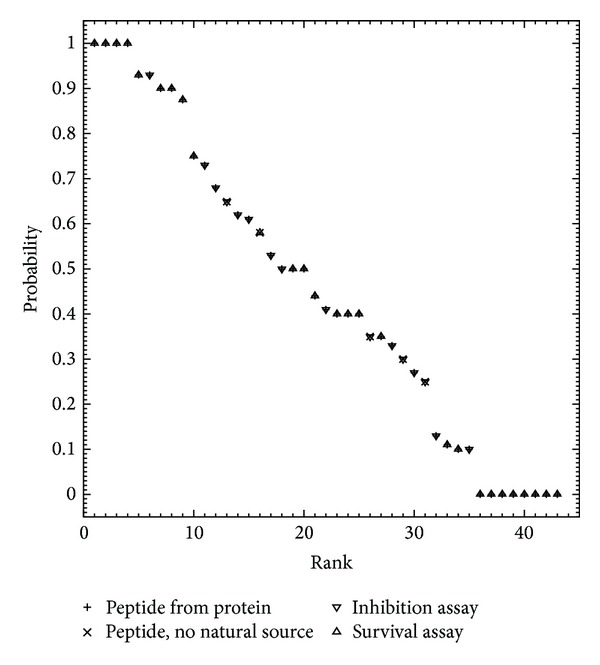
Probabilities expressing biological effects of B-cell epitope binding by antipeptide antibodies. Each data point is plotted as a pair of superposed symbols for immunogen type (peptide either from protein or with no natural source) and B-cell assay type (inhibition or survival). Data points are ranked in order of decreasing probability as in Table 1.

**Figure 3:**
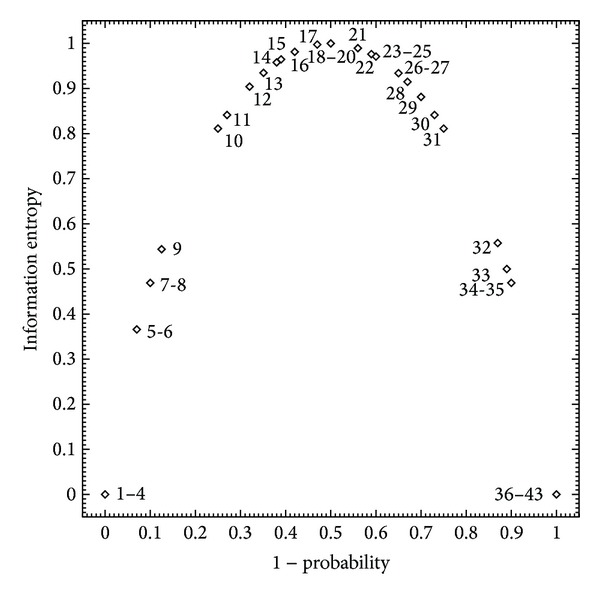
Information entropies for quantitative biological effects of antipeptide antibody binding, calculated from probabilities in Figure 2 using (8). Data points are labeled by probability rank as in Figure 2 and Table 1.

The above-mentioned subset comprised records pertaining to B-cell assays for which the antibodies were elicited against peptide epitopes whose sequences either were conceptually derived from cognate protein antigens (38 records) or had no known natural source (five records, ranked 13, 16, 26, 29, and 31 in Table 1 and Figure 2). For these records, all the assayed antibodies were polyclonal, and the B-cell assay type in each case was either neutralization/inhibition of antigen activity (16 records) or survival after challenge (27 records), with other assay types unrepresented. As all the retrieved quantitative data were thus found expressed as percentages (i.e., of either inhibition or survival), these were converted into fractional form to obtain values of the empirical quotient *q* in (3) and were interpreted accordingly as probabilities (Figure 2), for which corresponding values of the information entropy *H* were calculated in bits using (8) (Figure 3). The obtained values of *q* included both zero (eight records) and unity (four records), for which the assay type was neutralization/inhibition of antigen activity in all cases, as well as half unity (three records); hence, the calculated values of *H* ranged from zero to unity with the latter representing a much smaller minority than the former, such that maximally informative data were much fewer than minimally informative data. However, although the minimally informative data correspond to *H* values of zero, they nonetheless point to the possibility of modifying experimental conditions (e.g., antibody concentration [Ab]) in order to yield new data that are more informative. In particular, *q* values of zero and unity, respectively, suggest that more informative data might be obtained by either increasing or decreasing [Ab] so as to bring *q* closer to half unity (e.g., in accordance with (4) through (7)), with the prospect of such improvement being more generally conceivable where [Ab] would be decreased.

### 3.2. Potential Benchmark-Data Sources

Table 2 presents the breakdown of the 3996 retrieved IEDB records on biological effects mediated by antipeptide antibodies, in relation to B-cell assay type (defined by experimental method/technique) and including the counts of records curated as containing either positive or negative data (noting again that all IEDB records, regardless of actual quantitative-data content, are curated as such). The entire set of records thus found represents the evolving repertoire of potential benchmark-data sources for B-cell epitope prediction relevant to antibody-mediated biological effects.

On inspecting the record counts, the positive data clearly outnumber the negative data for all assay types other than enhancement/activation of antigen activity (in which case the positive and negative data counts are nearly equal). This observation would be consistent with an underlying publication bias towards underreporting of negative results, which seems plausible in view of the widely acknowledged difficulty of accurately generating positive B-cell epitope predictions [20, 54]. Such bias is potentially problematic where the published data could be misleading as basis for the development and benchmarking of computational tools for B-cell epitope prediction. This is especially important where the data fail to reflect trends towards negative outcomes of immunization (e.g., failure either to induce the production of antipeptide antibodies in the first place or of such antibodies to cross-react with antigenic targets in a manner that produces functionally relevant biological effects).

As regards assay-outcome units of measurement (in the IEDB assay-record data field named “Assay Type Units”), these are explicitly specified only for the IEDB assay types named “Neutralization/Inhibition of Antigen Activity,” “Protection After Challenge,” and “Survival After Challenge.” (“Survival After Challenge” is apparently subsumed under “Protection After Challenge” in the B Cell Assay Tree as shown in Figure 1, although the latter assay-type name was actually specified in records for which protection is against outcomes other than death, such as nonlethal infection or toxicity.) Furthermore, actual data values are often missing even where units of measurement are specified. This clearly indicates the potential for curation of quantitative data for the said assay types, although the vast majority of records for these types currently contain only qualitative data. As to all the other assay types, each of these conceivably could be cast as a quantitative assay type by specifying appropriate assay-outcome units of measurement. Towards this end, currently unspecified units of measurement could be specified by analogy to those assays for which the units of measurement are already provided; for instance, outcomes could be expressed as percentages in all cases, such that conversion of the percentages to corresponding fractions yields values of the empirical quotient *q* in (3).

Most of the assay types lacking units of measurement thus could be cast as quantitative by analogy to survival after challenge, which is expressed as survival [%]. This could be accomplished by defining each outcome as a proportion of individuals in a population that manifest a particular antibody-mediated effect (e.g., maintenance or change in health or immune status), as is arguably applicable to protection from fertility, reduction of disease after treatment, exacerbation of disease after treatment, hypersensitivity, and induction of tolerance. Likewise, antibody-dependent cellular cytotoxicity and complement-dependent cytotoxicity could be expressed as the proportion of target cells lysed. Moreover, Ab-dependent phagocytosis/opsonization might be quantitatively expressed as the proportion of either viable phagocytes observed performing phagocytosis or particulate targets (e.g., microbial cells) internalized by the phagocytes. This could be defined as being in excess of some antibody-free basal level (e.g., corresponding to negative controls with particulate targets devoid of coating antibody) and relative to an empirically determined maximum level (e.g., corresponding to positive controls for which the particulate targets are opsonized with saturating levels of coating antibody). Ig-mediated histamine release also might be expressed as the proportion of activated histamine-releasing cells (e.g., mast cells or basophils for which exocytosis is observed) or the fraction of histamine released by such cells, again in excess of some antibody-free basal level and relative to either a theoretical or an empirically determined maximum level (e.g., 100% occurrence of exocytosis among viable cells or maximum amount of histamine thus released).

Certain variations of the above-mentioned assay types also might be cast as quantitative by subsumption under neutralization/inhibition of antigen activity, which is expressed as inhibition [%]; for example, interference with Ig-mediated histamine release might be expressed as inhibition [%] by antibody (e.g., antipeptide IgG that binds antigen to prevent cross-linking of Fc*ϵ*RI-associated IgE on effector cells such as mast cells and basophils). Additionally, inhibition of Ab biological activity (e.g., inhibition of antibody-mediated biological effects by peptide antigen) likewise could be expressed quantitatively as inhibition [%] by peptide antigen (e.g., for experiments demonstrating the immunologic specificity of biological effects mediated by antipeptide antibodies). On another related note, the various assays already subsumed under neutralization/inhibition of antigen activity are all conceivably amenable to quantitation as inhibition [%]. As a case in point, antibody-mediated inhibition of cytopathic effect (CPE) among virus-infected cells can be expressed as the proportion of target cells remaining CPE-free despite inoculation with a quantity of virions producing CPE in 100% of target cells at zero antibody concentration. Further extending the concept of effects on target cells, even antibody-mediated hemagglutination inhibition (HI) could be expressed as the proportion of erythrocytes that remain unassociated; in practice, HI assays might be performed in order to determine the conditions (e.g., on the basis of HI titers) corresponding to 50% unassociated erythrocytes.

**Figure 4:**
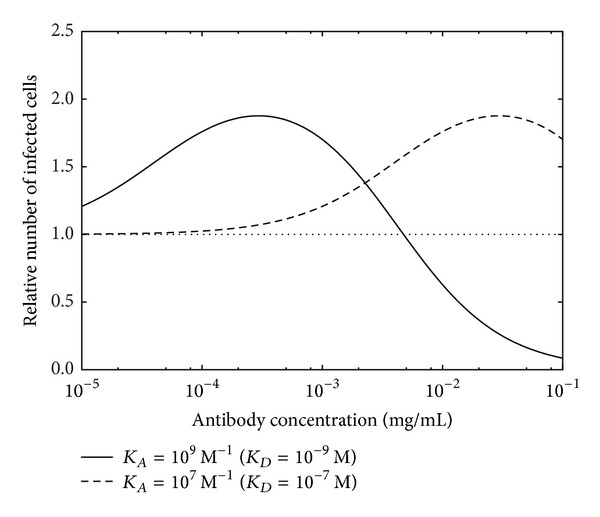
Antibody-dependent enhancement of infection as mathematically modeled for HIV in cultures of U937 monocytoid cells [17] in the limit of low multiplicity of infection. Relative number of infected cells is set to unity (represented by dotted baseline) for zero antibody concentration. Curves are shown for two different antibody affinity levels expressed in legend as association constant (and equivalent dissociation constant in parentheses). Enhancement could be expressed as a fraction of the maximum enhancement (i.e., at the peak of the curve), with zero enhancement at zero antibody concentration.

As regards Ab enhancement/activation of antigen activity, this might be quantified in various system-dependent ways. For instance, it might be expressed as the fraction of activated enzyme for a simple two-state model of antibody-mediated allosteric enzyme activation, or as a relative excess of infected cells in cases of antibody-mediated enhancement of infection. The latter situation is exemplified by the nonlinear relationship typically observed between enhancement/activation and antibody concentration, with enhancement of infection tending to occur maximally at some optimum antibody concentration (e.g., with infection of CD4-positive monocyte-like cells by HIV [17], as shown in Figure 4). In such cases, enhancement (e.g., defined as infection in excess of a baseline level at zero antibody concentration) could be expressed as a fraction of its maximal value at the optimum antibody concentration.

Hence, all IEDB B-cell assay types might be cast as quantitative to express data in a manner consistent with (3). However, even data thus expressed are of questionable value for benchmarking B-cell epitope prediction within a dose-response framework if unaccompanied by key contextual data, most especially on antibody concentration in view of (4) through (7). Currently, IEDB B-cell assay records lack data fields specifically designated for representing antibody concentration and many other data (e.g., antigen concentration and temperature) that are potentially important as input for B-cell epitope prediction; in principle, such data could be embedded in the text of the existing data field for assay comments, but this practice would be potentially problematic as regards data standardization.

### 3.3. Future Directions

Given the paucity of IEDB records containing explicitly specified curated quantitative data suitable for benchmarking B-cell epitope prediction to produce antipeptide antibodies that mediate biological effects, large-scale accumulation of these data is imperative for further development of B-cell epitope prediction methods. If this is to support biomedical applications such as the design of peptide-based vaccines, the data should represent dose-response relationships in a manner that is informative in the sense of having a positive information entropy (e.g., according to (8)) and being sufficiently qualified in terms of antibody concentrations and possibly other relevant parameters (e.g., antigen concentrations and temperature).

The numerical discrepancy between all records (3996) and those containing quantitative data (43) points to the possibility of increasing the latter via data curation. Standard IEDB curation practice includes attempts to extract quantitative data from sources in published literature [12], which is more straightforward to accomplish where the said data are written in the main text or in tables rather than depicted in graphs. Among data in graphical form, those representing proportions of populations (e.g., surviving fractions of populations in Kaplan-Meier survival curves) may be more readily ascertained than those representing continuous variables (e.g., inhibition of enzyme activity), particularly where population sizes are so small such that values corresponding to discrete numbers of individuals may be readily discerned. Alternatively, numeric values of graphically depicted data might be obtained through correspondence with authors in certain cases (still in accordance with standard IEDB curation practice [12]), especially for more recently published literature.

At a deeper level, the apparent numerical superiority of qualitative-only over quantitative IEDB B-cell assay records might be at least partially mitigated or even reversed in the long run by curation of data as quantitative rather than qualitative-only. This is feasible within the present IEDB framework only to the extent that units of measurement are already defined for particular assays, which is a limitation that could be overcome by defining such units as outlined in the immediately preceding Section 3.2, in order to accommodate specialized forms of quantitative data (e.g., on Ab-dependent phagocytosis/opsonization and on enhancement/activation of antigen activity). Nevertheless, available qualitative-only data may be viewed as guides to the further acquisition of quantitative data by means of specific experimental approaches. In particular, positive qualitative-only data might justify confidence in the feasibility of obtaining corresponding quantitative dose-response data, whereas negative qualitative-only data might anticipate difficulties in obtaining such quantitative data (e.g., without first increasing antibody concentrations used in the assays, which itself may still fail to demonstrate dose-response behavior under physically realizable conditions if antibody affinity is exceedingly low).

The above proposed measures could be applied to the curation of newly acquired data and possibly also to the recuration of data that are already within IEDB, in line with the goal of increasing the body of quantitative dose-response data for benchmarking B-cell epitope prediction. However, because minimally informative quantitative data (i.e., with zero information entropy, which corresponds to either undetectable or maximal responses) by themselves are inadequate as bases for delineating dose-response relationships (e.g., using (4) through (7)), such data are little if any more informative than qualitative-only data (which, as already discussed, can serve as guides to acquiring more informative quantitative data). At best, minimally informative quantitative data define lower and upper limits on a dosage range over which a dose-response relationship might be observed. For instance, in a quantitative assay for neutralization/inhibition of antigen activity wherein the antibody-mediated effect is a strictly monotonically increasing function of antibody concentration, a zero (i.e., baseline) response at some antibody concentration coupled with a maximal response at a higher antibody concentration together suggest that an antibody dose-response relationship might be observed somewhere between the two antibody concentrations. In such a case, the median effective antibody concentration may be provisionally estimated (e.g., as the geometric mean of the two antibody concentrations if they define the endpoint of a titration experiment employing serial antibody dilutions), whereas the actual value of the median effective antibody concentration would be maximally informative data (i.e., with an information entropy of one bit according to (8)). Combinations of minimally informative quantitative data might thus yield estimates of quantitative dose-response data that are useful for benchmarking B-cell epitope prediction, although this purpose still would be best served by accurately determined maximally informative quantitative data.

To appropriately highlight maximally informative quantitative dose-response data, these could be explicitly specified as the numeric values for outcomes of quantitative B-cell assays, insofar as each of these values would represent an overall dose-response relationship. Where a median effective dose (e.g., of antibody or antibody-blocking peptide antigen) can be defined in the context of a B-cell assay, it is the most representative dose for the underlying dose-response relationship in that it is an unbiased threshold or cut point for dividing the dose-response curve into low- and high-dose regimes. This role of the median effective dose is analogous to that of the dissociation constant (or, equivalently, of its reciprocal the association constant), whose numeric value is explicitly specified in IEDB B-cell assay records as a measure of antibody affinity for various quantitative assays relating to characterization of antibody binding. The value of the dissociation constant itself often may be regarded as a median effective concentration, at which exactly half of B-cell epitopes are antibody-bound (e.g., according to (4)). Presently, the IEDB curation guidelines provide for bulk curation of dose-response data obtained from a series of related assays wherein only dose is varied, with a single numeric value curated as representing the entire series, but this value is of the highest documented response in the series [12] rather than the median effective dose. Nevertheless, any available data on the median effective dose could be embedded within the existing data field for assay comments, so as to facilitate future assembly of benchmark datasets from such maximally informative data.

The special status of median effective doses as maximally informative data suggests the prospect of revising the IEDB curation guidelines in order to specify the value of the median effective dose (rather than that of the highest recorded response) as representing bulk-curated dose-response data. However, a more generally applicable approach to curating quantitative dose-response data would be to continue expressing actual response magnitudes as quantitative data (e.g., in terms of inhibition [%] and survival [%]) while explicitly qualifying these with respect to the conditions (of antibody concentration, antigen concentration, temperature, etc.) under which they were observed. This would adequately capture even observations not forming part of a dose-response series (e.g., where the extent of an antibody-mediated biological effect has been measured only for a single antibody concentration). As has already been indicated in the immediately preceding Section 3.2, this approach could be adopted within the present IEDB framework by specifying the conditions as data embedded in the existing data field for assay comments, albeit without the benefit of data standardization that otherwise could be achieved by introducing additional data fields. Yet, the alternative of adding such data fields would clearly be much more difficult to fully conceptualize and implement considering the current size and complexity of IEDB. Furthermore, the current practice of bulk-curating quantitative dose-response data as a single representative value serves to maintain the relative compactness of IEDB, but it nonetheless still facilitates the identification of published dose-response data that are represented as bulk-curated records in IEDB. This directs investigators to the original published sources from which entire sets of dose-response data might be extracted and incorporated into benchmark datasets for B-cell epitope prediction (e.g., for benchmarking with continuous rather than dichotomous data, according to (1) through (7)).

In line with the preceding considerations, an antibody-mediated biological effect might be expressed as an apparent change in the median effective dose (e.g., median lethal dose or median infective dose) of a particular causative agent (e.g., toxin or pathogen) as a function of antibody concentration; accordingly, the median effective dose might be apparently increased in the presence of antibody relative to an antibody-free baseline reference system. Numeric values of the baseline and apparently increased median effective doses in conjunction with the antibody concentration at which the apparent increase is observed could enable B-cell epitope prediction and benchmarking thereof, for example, where the ratio of the baseline to the apparently increased doses is estimated as the complement of the antibody-bound antigen fraction (e.g., by means of (4)). This approach is potentially applicable to assay types including survival after challenge and protection after challenge.

Ultimately, the most crucial determinant of the availability of benchmark data is their actual generation in the first place. Experimentalists thus could greatly contribute to the further accumulation of informative benchmark data by generating dose-response data at or near half-maximal response levels, expressing antibody-mediated effects as apparent concentration-dependent changes in median effective doses of particular causative agents wherever possible. This demands explicit specification of antibody concentrations in molar or equivalent terms rather than incommensurable arbitrary units (e.g., based on titers operationally defined only for a particular immunoassay protocol).

With further accumulation of quantitative data suitable for benchmarking B-cell epitope prediction as regards biological effects of antipeptide antibodies, rigorous validation would be increasingly enabled for B-cell epitope prediction tools to support the rational design of peptide-based immunogens relevant to the development of novel pharmaceutical products. (In particular, the said products would include peptide-based vaccines and prophylactic or therapeutic antipeptide antibodies including antidotes to other peptidic pharmaceutical agents.) By the same token, the new data could provide the basis for developing such B-cell epitope prediction tools via machine-learning techniques (e.g., using artificial neural networks and support vector machines) with emphasis on predicting biological outcomes of antibody binding as continuous rather than dichotomous variables.

The present work thus further clarifies the challenges of developing B-cell epitope prediction and how these might be overcome, particularly for vaccines and other biomedical applications. As noted in previous work, B-cell epitope prediction encompasses diverse phenomena ranging from mechanistically very simple cases (e.g., antibody-mediated enzyme inhibition in vitro) to high-level biological processes (e.g., of antibody-mediated immunity to infection) [9]. This suggests that B-cell epitope prediction can be systematically developed through iterative refinement extending its applicability to increasingly complex systems, towards the long-term goal of effecting biological outcomes that advance global health [10]. Accordingly, linear (i.e., continuous, rather than discontinuous) B-cell epitopes are the main concern of B-cell epitope prediction, as they can be operationally defined (i.e., as peptide sequences) even in the context of polyclonal antibody responses relevant to vaccine design [55]. Notwithstanding the importance of discontinuous B-cell epitopes (e.g., as virus neutralization epitopes [54]), progress in B-cell epitope prediction is thus currently a much more realistic prospect for linear rather than discontinuous epitopes, insofar as empirical confirmation of epitope-specific targeting is readily feasible with antipeptide antibodies directed to linear epitopes [55]. Consequently, application of information entropy to B-cell epitope prediction was initially outlined in preliminary form [56] and much more extensively elaborated in the present work vis-a-vis the body of pertinent data currently available via IEDB, within the practically meaningful context of prospective biomedical applications with due emphasis on pharmaceutical product development.

Still, B-cell epitope prediction conceivably could be developed much further for translational research. As a case in point, severity of infectious disease is a function of many factors (e.g., genetic background and life history). Hence, dose-response data on pathogen exposure levels vis-a-vis epitope-specific antibody levels may be difficult to model on the basis of currently sparse data, especially for prediction of stochastic individual-level outcomes (e.g., death or survival of a host organism). Nevertheless, the ability to predict antibody-mediated biological effects even only in terms of statistical averages (i.e., of groups rather than individuals) is of practical value (e.g., in order to attain population-level resistance to epidemic spread of infectious disease [55]).

## 4. Conclusions

Quantitative dose-response data represent a more practically meaningful alternative to qualitative dichotomous data as basis for benchmarking B-cell epitope prediction particularly where antibody-mediated biological effects are the outcomes of interest, as exemplified by peptide-based vaccine design and similar efforts toward the production of antipeptide antibodies that cross-react with proteins and thereby modulate protein function. The said effects typically can be expressed in fractional form relative to context-dependent maximal effects, allowing for the evaluation of the Shannon information entropy where the effects may be interpreted in terms of underlying two-state probability distributions. According to this scheme, maximally informative data correspond to half-maximal effects while minimally informative data correspond to either zero or maximal effects; although the latter data are associated with zero information entropy, they may still be informative in the sense of suggesting possible changes in experimental conditions to yield more informative data (e.g., by adjusting antibody concentrations to approach half-maximal levels of antibody-mediated effects). The present paucity of such informative dose-response data, particularly as observed in IEDB, thus conceivably could be addressed through large-scale generation and curation of data on antibody concentrations vis-a-vis quantitative effects of antibody binding, expressing the antibody-mediated effects as apparent concentration-dependent changes in median effective doses of particular causative agents such as toxins and pathogens wherever applicable. This could better enable B-cell epitope prediction to better support the development of novel pharmaceutical products such as peptide-based vaccines and antipeptide antibodies including antidotes to other pharmaceutical agents.

## Figures and Tables

**Table 1:** IEDB data on quantitative biological effects of binding by antipeptide antibodies.

Rank	BCell ID	Epitope ID	PubMed ID	Ref. number
1	4387	59318	15530682	[23]
2	1271872	42596	16713037	[24]
3	1478698	43317	17942539	[25]
4	1787548	135799	18725625	[26]
5	1031769	60116	1700835	[27]
6	1346451	62348	1730474	[28]
7	1464052	6474	16154668	[29]
8	1464085	58344	16154668	[29]
9	1479673	27725	8806185	[30]
10	1036155	48765	1695255	[31]
11	1844776	148424	9764364	[32]
12	1844777	147600	9764364	[32]
13	1784451	134549	2140594	[33]
14	1342494	6402	9234808	[34]
15	1346452	11824	1730474	[28]
16	1270835	22873	16545605	[35]
17	1342371	10069	9234808	[34]
18	1347617	43152	1377851	[36]
19	82	14686	15710332	[37]
20	1651107	108291	19356802	[38]
21	1032259	41770	9453605	[39]
22	1346453	11817	1730474	[28]
23	1246340	52790	2473217	[40]
24	1244600	20463	9795391	[41]
25	1464091	57812	16154668	[29]
26	9202	62340	11376846	[42]
27	1651118	108482	19356802	[38]
28	1844778	147559	9764364	[32]
29	9199	24243	11376846	[42]
30	1844774	148481	9764364	[32]
31	9196	42264	11376846	[42]
32	1844779	148231	9764364	[32]
33	1246395	31454	2473217	[40]
34	1246390	12540	2473217	[40]
35	1844780	147543	9764364	[32]
36	1246339	56951	2473217	[40]
37	1209202	44865	1701079	[43]
38	1209204	70682	1701079	[43]
39	1209209	60544	1701079	[43]
40	1209236	6743	1701079	[43]
41	1244602	2773	9795391	[41]
42	1464078	1531	16154668	[29]
43	1464093	59151	16154668	[29]

**Table 2:** IEDB B-cell assay record counts of selection in Figure 1.

Method/technique	Total	Positive	Negative
Ab-dependent Phagocytosis/opsonization	**162 **	120	42
Antibody-dependent cellular cytotoxicity	**42 **	34	8
Complement-dependent cytotoxicity	**63 **	34	29
Enhancement/activation of antigen activity	**21 **	10	11
Exacerbation of disease after treatment	**185 **	93	92
Hypersensitivity	**20 **	14	6
Ig-mediated histamine release	**8 **	7	1
Induction of tolerance	**66 **	51	15
Inhibition of Ab biological activity	**17 **	12	5
Neutralization/inhibition of antigen activity	**1995 **	1337	658
Protection after challenge	**858 **	499	359
Protection from fertility	**79 **	50	29
Reduction of disease after treatment	**125 **	96	29
Survival after challenge	**325 **	211	114
All of the above methods/techniques	**3966 **	2568	1398
